# The Development of Multiple Periprosthetic Joint Infections in Conjunction With Ibrutinib Therapy

**DOI:** 10.7759/cureus.20639

**Published:** 2021-12-23

**Authors:** Swathi Muttana, Christopher Solowiej Singh, Harim Kim, Christopher J Smith, Miriam B Michael

**Affiliations:** 1 Internal Medicine, American University of Antigua, New York, USA; 2 Internal Medicine, University of Maryland Medical Center Midtown Campus, Baltimore, USA; 3 Infectious Disease, University of Maryland Medical Center, Baltimore, USA; 4 Internal Medicine, Howard University, Washingon DC, USA; 5 Internal Medicine, University of Maryland, Baltimore, USA

**Keywords:** waldenströms macroglobulinemia, joint aspiration, joint infection, ibrutinib, periprosthetic joint infection

## Abstract

Periprosthetic joint infections (PJI) can be subcategorized into acute postoperative infections, occurring within three months of implantation, and delayed onset infections, occurring after three months of implantation. PJIs can be caused by numerous infectious etiologies. Here, we describe a unique case of a patient with a history of bilateral shoulder and knee replacements over five years. The patient received a diagnosis of Waldenströms macroglobulinemia five years before her admission but deferred ibrutinib treatment until one year before her admission. We believe that the timeline coincides with the development of multiple PJIs secondary to ibrutinib therapy. The patient presented with bilateral shoulder and knee pain and swelling, following a flu-like illness that had resolved one year before the admission. Her joint symptoms did not subside along with the remaining flu-like symptoms. Initially, her symptoms served as clues to the diagnosis; however, the diagnosis was finally made and supported by joint aspiration. The patient was treated with vancomycin 1.25 g in sodium chloride 0.9% 250 mL intravenous piggyback every 24 hours for the treatment of PJI and oral daptomycin 500 mg daily for six weeks as prophylaxis for PJI. In conclusion, physicians need to consider the development of PJIs when prescribing immunosuppressive therapy, as well as an early diagnosis to prevent further complications.

## Introduction

Periprosthetic joint infection (PJI) is a severe complication after arthroplasty, which is associated with pain, prolonged hospitalization, need for additional surgeries, functional incapacitation, and even mortality [1]. Joint replacement is a life-enhancing procedure performed for millions of people worldwide each year. Successful joint replacement provides pain relief, restores function and independence, and improves the quality of life. While already a frequently performed procedure, the incidence of prosthesis implantation is expected to continually rise with total knees reaching one million in the next two years [2]. PJI occurs in 1-2% of primary and 4% of revision arthroplasties. Approximately two-thirds of PJI cases are caused by the intraoperative inoculation of microorganisms. Depending on microbial virulence, PJI can manifest either early (within the first four weeks of implantation) or with a delay (typically between three months and three years). Early infections manifest with clear local and systemic signs of inflammation and are predominantly caused by highly virulent pathogens (e.g., *Staphylococcus aureus*, streptococci, enterococci). Delayed infections present with more subtle symptoms such as joint pain and early loosening and are caused by low-virulent organisms (e.g., *Propionibacterium acnes*, *S. lugdunensis*) [3]. Immunocompromised patients are thought to be at higher risk for the hematogenous spread of infections.

## Case presentation

A 70-year-old female was diagnosed with Waldenström macroglobulinemia five years ago. For the first four years after the diagnosis, the patient did not receive any treatment for Waldenström macroglobulinemia. For the last year, the patient was being managed with ibrutinib 280 mg tablet orally daily. The patient underwent bilateral total knee arthroplasty (five years before presentation) and bilateral total shoulder arthroplasty (two and three years, respectively, before presentation), which were complicated by chronic multi-joint PJIs.

The patient reported a one-year history of pain bilaterally in her knees and shoulders, which she believed co-occurred with an influenza-like illness. However, her pain had persisted over the past year while all other symptoms had subsided. One month before the patient’s admission, she was seen by her outpatient orthopedic surgeon who aspirated bilateral shoulders and knees. Left shoulder aspiration revealed 4,356 white blood cells (WBCs) (99% polymorphonuclear leukocytes [PMNs]). Right shoulder aspiration revealed 14,750 WBCs (95% PMNs). Left knee aspiration revealed 8,002 WBCs (85.7% PMNs). Additionally, alpha-defensin and *Staphylococcus* panel were positive but the culture was negative. Right knee aspiration revealed 31,830 WBCs (86.3 % PMNs), with alpha-defensin and *Staphylococcus* positivity (Table 1). These findings were consistent with multi-joint PJI and were indicative of hematogenous spread.

**Table 1:** Findings of joint aspiration. WBC: white blood cell; PMN: polymorphonuclear leukocyte

	WBC count	PMNs in WBC count (%)	*Staphylococcus*positivity	Alpha-defensin positivity
Left knee	8,002	85.7%	+	+
Right knee	31,830	86.3%	+	+
Left shoulder	4,356	99%	Not reported	Not reported
Right shoulder	14,750	95%	Not reported	Not reported

Given that the patient was diagnosed with Waldenström macroglobulinemia five years before the initiation of ibrutinib therapy, had no history of PJI infections, and had developed multi-joint PJI, the multi-joint PJI likely occurred secondary to the ibrutinib therapy rather than secondary to an underlying hematologic malignancy.

The patient was admitted to the hospital due to concerns of multi-joint PJI. During the initial stages of the patient’s admission, she was anemic, for which she underwent several blood transfusions. Following each transfusion, she developed a fever, which was likely due to a transfusion reaction. The patient underwent aspiration of bilateral shoulders and explant of bilateral knees along with antibiotic spacer placement. For the treatment of the multi-joint PJI, the patient received vancomycin 1.25 g intravenously every 24 hours.

During her inpatient stay, she developed shortness of breath, for which she was placed on supplemental oxygen. A computed tomography angiogram revealed a pulmonary embolism, which was attributed to the patient’s immobilization. She was immediately placed on a therapeutic heparin infusion and then transitioned to a direct oral anticoagulant (DOAC) for three to six months. Upon discharge, the patient was placed on daptomycin 500 mg orally daily for six weeks as a prophylactic treatment for multi-joint PJI.

## Discussion

This case presents a patient with a five-year history of Waldenström macroglobulinemia who developed PJIs in bilateral shoulders and knees following a one-year course of ibrutinib treatment. Four joints concurrently undergoing PJI is a unique finding, and few cases have been reported [4]. These previously reported cases presented clinically similar to our patient, and the diagnosis was primarily made and confirmed via joint aspiration [4]. The patients were additionally managed initially with surgical intervention and aspiration, followed by antibiotics for treatment and prophylaxis [4].

One limitation of this case report is the inability to determine the exact cause of infection in our patient. The patient was diagnosed with Waldenström macroglobulinemia five years ago and was started on ibrutinib treatment one year ago. For patients with Waldenström macroglobulinemia, infection risk has been shown to be the highest in the year of the diagnosis and decreases over time [5]. An increased risk of infections is also a well-known adverse effect associated with ibrutinib, with the risk of infection being the highest in the first year after starting treatment [6-8]. Given the more recent initiation of ibrutinib treatment, we believe that immunosuppressive therapy predisposed the patient to the rare multi-joint PJI.

History

Clarifying the patient’s history including the history of prosthetic placement as well as oncologic history is important in diagnosing PJI [4]. Our patient had undergone prosthetic placements in bilateral knees due to osteoarthritis and in bilateral shoulders due to rotator cuff injuries, eliminating the correlation between her diagnosis of Waldenström macroglobulinemia. The patient additionally stated that the onset of inflammation and pain in all four joints co-occurred with influenza (not confirmed) in May 2020, indicating chronicity.

Diagnosis

To save the prosthesis and joint function, early diagnosis is favorable. There is no single test for diagnosis rather a combination of tests may be used to increase the accuracy of diagnosis. The European Bone and Joint Infection Society criteria are the most sensitive for diagnosing PJI compared to other criteria, as shown in Table 1 [4].

**Table 2:** Criteria for diagnosing periprosthetic joint infection. The diagnosis is confirmed if at least one of the four criteria is fulfilled. Derived from Li et al. [4].

Diagnostic test	Criteria	Sensitivity (%)	Specificity (%)
Clinical features	Sinus tract or visible purulence	20–30	100
Histology in periprosthetic tissue	Acute inflammation in periprosthetic tissue	95–98	95–98
Leukocyte count in synovial fluid	>2,000/µL leukocytes or >70% granulocytes	93–96	93–96
Microbiology (culture)	Synovial fluid or tissue samples or sonication fluid (≥50 CFU/mL)	60–80	97
		70–85	92
		85–95	95

Joint aspiration

Joint aspiration and culture are often used to confirm the diagnosis of PJI, and studies have shown that it is both sensitive and specific in patients at moderate-to-high risk. Direct aspiration revealed an overall sensitivity of 81% (71-88%) and a specificity of 90% (85-93%) in a previous study [4]. This method allows for susceptibility testing of relevant organisms, guiding antibiotic therapy. The diagnosis in our patient was based on joint aspiration results (Table 2). Before the patient’s hospital admission, she was seen in the outpatient setting in which several synovial aspirations were obtained. Throughout the patient’s admission, her serum WBCs were elevated.

Treatment, prophylaxis, and complications

Our patient required orthopedic surgical intervention in each of the four joints. For this, aspiration of bilateral shoulders and explant of bilateral knee arthroplasty with the placement of antibiotic spacers was done. During in-patient hospitalization, the patient was managed for PJI using intravenous vancomycin. Finally, she was discharged using daptomycin as prophylactic treatment. During her hospitalization, her low hemoglobin levels required several blood transfusions to replete to stable levels. Following each transfusion, the patient’s vitals were positive for fever spikes. Additionally, she developed a pulmonary embolism which was likely attributed to immobilization.

Ibrutinib

The patient described in this study was being managed for Waldenström macroglobulinemia using ibrutinib, which we believe played a role in the development of PJI. Ibrutinib is an oral chemotherapeutic agent used in the treatment of several cancers, including chronic lymphocytic leukemia, B-cell lymphoma, and Waldenström macroglobulinemia [6,9].

Ibrutinib targets Bruton tyrosine kinase (BTK), a member of the Tec family of non-receptor protein-tyrosine kinases [6]. BTK is an important signaling molecule in the B-cell antigen receptor pathway, as well as an intermediate of other downstream pathways such as the nuclear factor kappa B and nuclear factor of activated T-cells pathways [6,10]. Ibrutinib belongs to the Tec family of protein-tyrosine kinases which play an important role in several immune system cells [7]. Loss of BTK expression is seen in the genetic condition X-linked agammaglobulinemia (XLA) and results in an absence of circulating B-cells and an immunocompromised state [7]. Ibrutinib acts by irreversibly inhibiting BTK by covalently binding to a cysteine residue on the ATP-binding pocket in the active site [7]. Blockade of BTK by ibrutinib has been reported to promote apoptosis and inhibit cell signaling and proliferation in cancerous cells making it an effective treatment for various cancers [11]. Although the main cellular target of ibrutinib is BTK, it has also been shown to exert some level of activity on at least 19 other kinases [11].

The adverse effects of ibrutinib have been well documented and include neutropenia, thrombocytopenia, anemia, atrial fibrillation, increased bleeding risk, diarrhea, arthralgias, hypertension, and infections [4,6,7]. Ibrutinib therapy causes an immunocompromised state and increases the risk of infections [6,8,10,12]. The greatest risk of infection occurs early, specifically in the first months after initiating ibrutinib treatment [6-8]. Although the loss of BTK function in ibrutinib-treated patients parallels that seen in XLA, it is worth noting that patients treated with ibrutinib tend to have infections from a larger variety of infectious organisms when compared with XLA patients [6]. It has been suggested that this may be due to ibrutinib’s off-target inhibition of non-BTK Tec family proteins [6]. Ibrutinib appears to affect the immune system in several ways leading to an immunocompromised state. It impairs an interleukin-2-inducible T-cell kinase which plays an important role in immune function [13]. In addition, ibrutinib impairs the antibody-dependent cell-mediated cytotoxicity of natural killer cells [13]. In addition, ibrutinib has been shown to cause functional deficits in neutrophils and impair neutrophil oxidative burst [9]. Finally, it has been shown to interfere with macrophage phagocytosis [8]. With many mechanisms implicated, the immunocompromised state seen in patients on ibrutinib is likely multifactorial. Additionally, ibrutinib should be withheld three to seven days before and following any surgery [13].

A previous study followed patients who were receiving ibrutinib for five years. Overall, 11.4% of these patients developed serious infections mainly during the first year of treatment. Of this 11.4% of the patients, 53.3% developed invasive bacterial infections, while 37.2% developed invasive fungal infections. Neutropenia was the most common cause of bacterial infection, and the most common bacterial pathogen was *Staphylococcus aureus*. Majority of the fungal infections that developed included invasive *Aspergillosis*, *Pneumocystis jirovecii* pneumonia, *Candida albicans*, and *Crypotococcosis *[1]. Regarding invasive fungal infections, there has been evidence showing impairment solely of the innate while sparing adaptive immunity [6].

Another study revealed four patients who developed total knee arthroplasty-related PJI following the initiation of chemotherapy for hematologic malignancy [4]. The chemotherapeutic agents used included ibrutinib, bortezomib, and/or lenalidomide with/without dexamethasone. This study expressed concern for profound immunosuppression resulting in the development of PJI. This suggests that immunosuppressive therapy may have contributed to the development of PJI in this patient.

## Conclusions

We have reported the case of a 70-year-old female with a history of bilateral shoulder and knee replacements and a diagnosis of Waldenström macroglobulinemia who developed concurrent PJIs in each of her four prosthetic joints within one year of initiating ibrutinib therapy. We attributed the occurrence of PJI to ibrutinib due to the onset of her symptoms roughly around the same time as the initiation of the immunosuppressive therapy. We believe that PJI in our patient can be attributed to her immunosuppressive therapy. Physicians should consider the development of PJIs when prescribing immunosuppressive therapy as well as the use of prophylaxis. Moreover, patients should be routinely monitored for early diagnosis to prevent further complications. We hope to educate both hematologist-oncologists and orthopedic surgeons in considering the risk of PJIs in joint arthroplasty in conjunction with immunosuppressive therapy, such as ibrutinib.
